# Toward the Positional Cloning of *qBlsr5a*, a QTL Underlying Resistance to Bacterial Leaf Streak, Using Overlapping Sub-CSSLs in Rice

**DOI:** 10.1371/journal.pone.0095751

**Published:** 2014-04-21

**Authors:** Xiaofang Xie, Zhiwei Chen, Jinliang Cao, Huazhong Guan, Degong Lin, Chunlan Li, Tao Lan, Yuanlin Duan, Damei Mao, Weiren Wu

**Affiliations:** 1 Key Laboratory of Ministry of Education for Genetics, Breeding and Multiple Utilization of Crops, Fujian Agriculture and Forestry University, Fuzhou, China; 2 Fujian Provincial Key Laboratory of Crop Breeding by Design, Fujian Agriculture and Forestry University, Fuzhou, Fujian, China; New Mexico State University, United States of America

## Abstract

Bacterial leaf steak (BLS) is one of the most destructive diseases in rice. Studies have shown that BLS resistance in rice is quantitatively inherited, controlled by multiple quantitative trait loci (QTLs). A QTL with relatively large effect, *qBlsr5a*, was previously mapped in a region of ∼380 kb on chromosome 5. To fine map *qBlsr5a* further, a set of overlapping sub-chromosome segment substitution lines (sub-CSSLs) were developed from a large secondary F_2_ population (containing more than 7000 plants), in which only the chromosomal region harboring *qBlsr5a* was segregated. By genotyping the sub-CSSLs with molecular markers covering the target region and phenotyping the sub-CSSLs with artificial inoculation, *qBlsr5a* was delimited to a 30.0-kb interval, in which only three genes were predicted. qRT-PCR analysis indicated that the three putative genes did not show significant response to the infection of BLS pathogen in both resistant and susceptible parental lines. However, two nucleotide substitutions were found in the coding sequence of gene LOC_Os05g01710, which encodes the gamma chain of transcription initiation factor IIA (TFIIAγ). The nucleotide substitutions resulted in a change of the 39^th^ amino acid from valine (in the susceptible parent) to glutamic acid (in the resistant parent). Interestingly, the resistant parent allele of LOC_Os05g01710 is identical to *xa5*, a major gene resistant to bacterial leaf blight (another bacterial disease of rice). These results suggest that LOC_Os05g01710 is very possibly the candidate gene of *qBlsr5a*.

## Introduction

Plant disease resistance is crucial for the security of crop production. Plant disease resistance can be classified into two categories: qualitative resistance and quantitative resistance. The former is controlled by single resistance (R) genes, while the latter is controlled by multiple genes or quantitative trait loci (QTLs), each of which only contributes a fraction to the resistance [1]. As qualitative resistance has much simpler genetic basis and usually exhibits large effect, efforts have been largely devoted to the study of R genes. At least 70 R genes have been cloned and some of them have been well characterized [2]. Most of the cloned R genes encode cytoplasmic receptor-like proteins characterized by an N-terminal nucleotide binding site (NBS), leucine-rich repeat (LRR) domain, leucine zipper (LZ), toll interleukine 1-receptor (TIR) or coiled-coil (CC) sequence [2]. In rice, more than 20 R genes have been cloned, such as *Pia* [3], *Pi36* [4], *Pib* [5], *Xa27* [6], *Xa1* [7] and *xa5* [8]. These studies have provided a wealth of information on the structure, function and evolution of R genes and have generated useful genetic materials for crop breeding.

The action of R genes, however, is generally race-specific. Therefore, they are apt to be quickly defeated by co-evolving pathogens [9]. In contrast, the multiple genes that control quantitative resistance are usually non-race-specific; they do not prevent infection but slow down pathogen development at the infection sites on the plant [10]–[12]. Therefore, quantitative resistance is much more durable and would be more favored in crop production. However, the molecular mechanism of quantitative resistance has been still unclear. Due to the small effect of each QTL and the influence of environment, it is difficult to clone quantitative resistance genes and characterize their molecular functions. To date, numerous disease resistance QTLs have been mapped in plants, but only a few of them with large effects have been isolated through positional cloning. In wheat, two major QTLs of disease resistance have been cloned. One is *Yr36*, which confers resistance to stripe rust owing to a single gene that encodes a kinase with a putative START lipid-binding domain [13]; the other is *Lr34*, which confers broad-spectrum and durable resistance owing to a single gene that encodes an ABC transporter [14], [15]. In rice, a major QTL conferring non-race-specific resistance to blast was found to be attributed to a single gene *pi21* [16], which encodes a proline-rich protein containing a putative heavy metal-binding domain and putative protein-protein interaction motifs [17]. By now, no disease resistance QTL of small effect has been cloned according to our knowledge.

Bacterial leaf streak (BLS) caused by *Xanthomonas oryzae* pv. Oryzicola is one of the most devastating diseases in rice. It occurs worldwide and is especially severe in southern China and other tropical and sub-tropical areas of Asia. Studies have indicated that BLS resistance in rice is quantitatively inherited [18], [19], and at least 13 QTLs conferring BLS resistance have been reported so far [19]−[21], but none of them has been cloned. Among the 11 QTLs mapped by Tang et al. [19], *qBlsr5a* on the short arm of chromosome 5 showed the largest effect, explaining ∼14% of phenotypic variation in the population of recombinant inbred lines used for the study. Subsequent studies confirmed the existence of *qBlsr5a* [22] and fine mapped the QTL to a region of ∼300 kb [23]. Although *qBlsr5a* has a relatively large effect, the BLS-resistance (lesion length) displayed typical quantitative variation following a normal distribution in the secondary F_2_ population used for fine mapping *qBlsr5a*, in which only the chromosomal segment harboring *qBlsr5a* was segregated [23]. Hence, *qBlsr5a* still belongs to a minor (or small-effect) QTL compared with the effect of environmental variation. In this study, we further narrowed down the interval and identified the candidate gene of *qBlsr5a*, approaching toward the positional cloning of the QTL.

## Materials and Methods

### Development of Overlapping Sub-CSSLs

The parental lines used by Tang et al. [19] for mapping BLS resistance QTLs were Acc8558 (or named DZ60, highly resistant to BLS) and H359 (highly susceptible to BLS), respectively. With Acc8558 as the donor parent and H359 as the recurrent parent, a chromosome segment substitution line (CSSL) named H359-BLSR5A, which only carried the resistant allele of *qBlsr5a* but none of other BLS-resistance QTLs from the donor parent, was previously developed by marker-assisted backcross breeding [22]. In this study, H359 and H359-BLSR5A were used as parents to produce a large F_2_ population.

According to Han et al. [23], *qBlsr5a* is located between SSR markers RM153 and RM159 (spanning ∼300 kb or 2.4 cM) on chromosome 5. Normally, we could take this interval as the target region for further fine mapping of the *qBlsr5a*. However, to avoid starting the fine mapping from a wrong interval, we reexamined the region. We found that although *qBlsr5a* was likely to be located between RM153 and RM159 as suggested by Han et al. [23], it still had a possibility to fall in an adjacent interval between RM159 and RM7029 (spanning ∼80 kb). Hence, we chose the region RM153-RM7029 (spanning ∼380 kb) as the target region for fine mapping.

The F_2_ seeds were soaked and pre-germinated, and then sown on seedling plates. At the seedling stage before transplantation, the two boundary markers of the target region were used to identify recombinant F_2_ plants, which all carried a recombined chromosome with the recombination point located within the target region. In other words, these plants all showed a homozygous genotype (i.e., either of the two parental genotypes) at one marker and the heterozygous genotype at the other marker. The recombinant seedlings were transplanted onto the field and the F_3_ seeds produced by them were harvested from individual plants separately.

The F_3_ seeds were sown in lines on seedling plates after pre-germination. One hundred seeds were sown per line. The two boundary markers of the target region were used to identify F_3_ seedlings that showed the genotype of one parent (say H359) at one marker and of the other parent (say H359-BLSR5A) at the other marker. In other words, these seedlings were homozygous recombinants for the target region. The homozygous recombinant seedlings from the same line constituted a sub-CSSL, which were transplanted onto the field for BLS resistance evaluation.

### Development and Analysis of Molecular Markers

Simple sequence repeats (SSR) markers in the target region were searched from the database Gramene (http://www.gramene.org/). To develop InDel markers, the publicly available rice genome sequences of indica cultivar 93-11 and japonica cultivar Nipponbare (http://www.gramene.org/) were compared to identify InDels using the online program Blast2 (http://blast.ncbi.nlm.nih.gov/Blast.cgi) and then primers for amplifying the InDel sequences were designed using the online program Primer3 (http://biotools.umassmed.edu/bioapps/primer3_www.cgi). Primers of the SSR and InDel markers were synthesized by Shanghai Sangon Biotechnology Company. Polymorphisms of the SSR and InDel markers between the two parents were tested by PCR. DNA was extracted from fresh leaves at the seedling stage using the CTAB method [24]. PCR amplification was conducted following Duan et al. [25]. PCR products were separated on 9% polyacrylamide denaturing gels and bands were visualized by silver-stain following Panaud et al. [26].

### Phenotyping and Genotyping of Sub-CSSLs

Following the method described by Tang et al. [19], plants at the active tillering stage were inoculated using the pricking inoculation method and the lesion length on each leaf was measured 20 days after inoculation. The pathogen isolate of *Xanthomonas oryzae* pv. Oryzicola used for inoculation was kindly provided by Prof. Guoying Chen of Huazhong Agricultural University. The resistance of each plant was indicated by the mean lesion length of three leaves and the resistance of each sub-CSSL was indicated by the mean lesion length of 10 plants. Meanwhile, the sub-CSSLs were genotyped using the polymorphic markers developed above, and the graphic genotype of each sub-CSSL was drawn.

### Gene Prediction and Sequence Analysis

Putative genes in the region of *qBlsr5a* were predicted by referring to the Rice Genome Annotation Project (http://rice.plantbiology.msu.edu/). Total RNA was extracted from leaves of the two parents using Trizol reagent (Invitrogen) and reversely transcribed into cDNA using Reverse Transcription Kit (Takara). The coding regions of the putative genes were amplified from the cDNA using PFU polymerase (Takara) and sequenced by Shanghai Sangon Biotechnology Company. DNA sequence comparison between the parents was performed using the BLAST program.

### Gene Expression Analysis

Expression of the predicted genes in the two parents and F_1_ in response to the infection of BLS pathogen was analyzed using real-time quantitative PCR (qRT-PCR). Leaves of similar age at the active tillering stage were inoculated with BLS pathogen (treatment) and sterile water (control), respectively. Each leaf was inoculated at three different sites as replicates. Total RNA extraction was performed at 6, 12, 24, 48 and 72 h after inoculation, respectively. For each leaf, three 1.0-cm sections each carrying an inoculation site were clipped and pooled as a sample for total RNA extraction. Only one pooled sample was used for each treatment. Each sample was assayed by qRT-PCR with three replicates using SYBR Premix Ex Taq (Takara). Actin gene was used as the internal control. A fold change of 2 (between treatment and control) was used as the cut-off value for identifying differential expression.

## Results

### Fine Mapping of *qBlsr5a*

**Figure 1:**
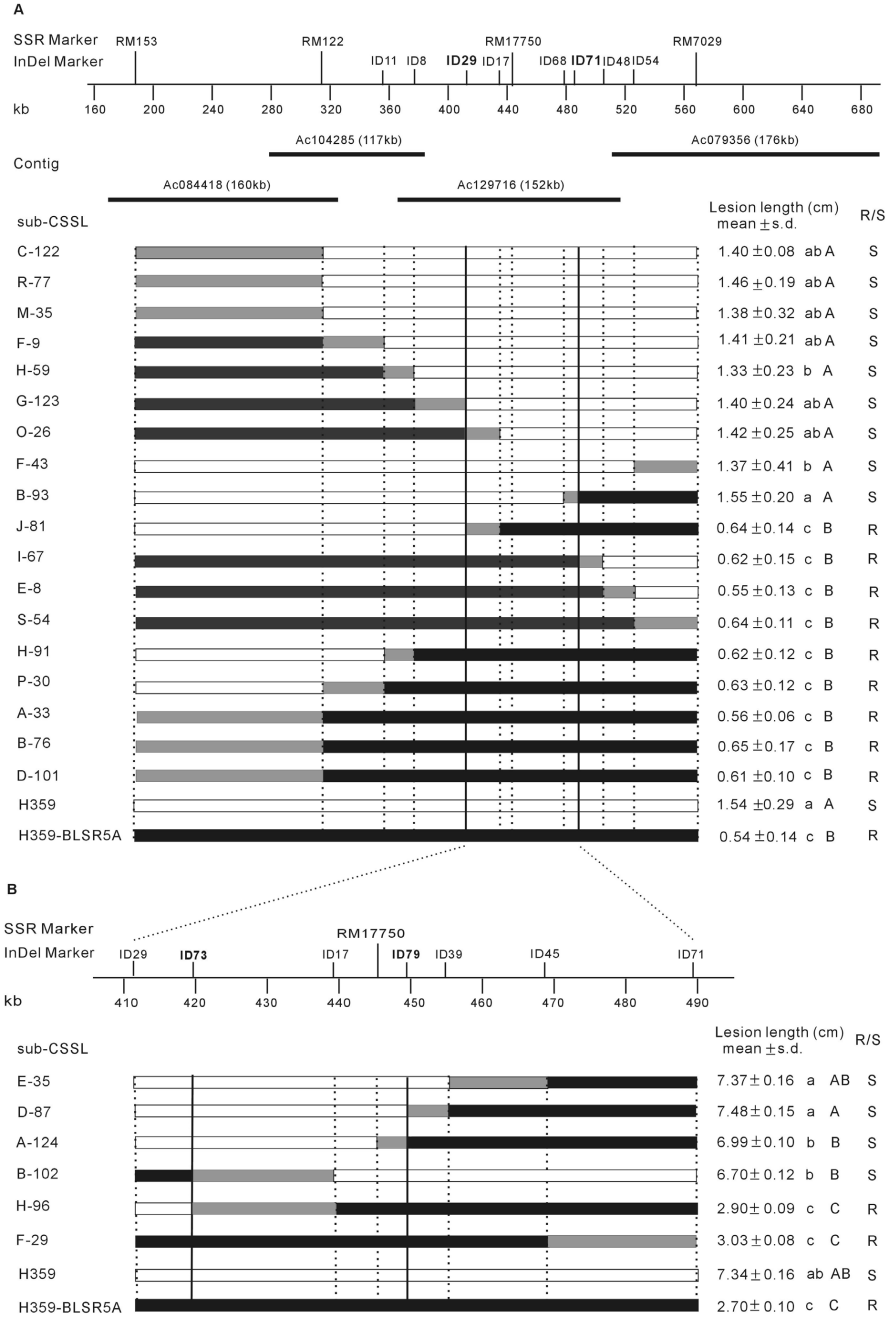
Physical map of the target region and graphical genotypes and resistance phenotypes of the sub-CSSLs. The open bars, solid bars and gray bars represent H359, H359-BLSR5A and recombined segments, respectively. The vertical solid/dotted lines indicate molecular marker positions. The lowercase and uppercase letters after the lesion length indicate statistical difference at 0.05 and 0.01 significance levels, respectively. R/S: resistant/susceptible to BLS.

A total of 2518 F_2_ seedlings were tested with the two markers RM153 and RM7029 delimiting the target region of *qBlsr5a*. Eighteen of the F_2_ seedlings were found to be the recombinants needed and therefore were transplanted onto the field. From the progeny (F_3_) lines of these 18 F_2_ plants, corresponding sub-CSSLs were developed, among which 10 sub-CSSLs exhibited the H359-BLSR5A genotype at RM153 but the H359 genotype at RM7029, while the other 8 sub-CSSLs just showed the opposite genotypes at these two markers (Figure 1A).

Inoculation experiment indicated that the average lesion lengths of the susceptible parent H359 and the resistant parent H359-BLSR5A were 1.54 cm and 0.54 cm, respectively, between which the difference was statistically significant at 1% level (Figure 1A). With the two parents as controls, the 18 sub-CSSLs could be clearly classified into two groups according to the results of ANOVA and Duncan’s test, with both 9 sub-CSSLs being susceptible and resistant to BLS, respectively (Figure 1A).

To narrow down the interval of *qBlsr5a*, we firstly chose two SSR markers (RM122 and RM17750) located approximately at the 1/3 and 2/3 points between RM153 and RM7029 to analyze the 18 sub-CSSLs. According to the genotypes of the sub-CSSLs at the four SSR markers (RM153, RM122, RM17750 and RM7029) and their phenotypes on BLS resistance (Figure 1A), it was found that the genotypes of lines F9, H-59, G-123, O-26, J-81, H-91 and P-30 in the interval between RM153 and RM122 contradicted their phenotypes. Therefore, this interval could be excluded and it could be deduced that *qBlsr5a* is located between RM122 and RM7029.

Subsequently, we developed eight InDel markers (ID11, ID8, ID29, ID17, ID68, ID71, ID48 and ID54; Table 1), which were approximately evenly distributed between RM122 and RM7029, to genotype the sub-CSSLs. The results indicated that the genotypes of lines O-26 and J-81 in the interval between RM122 and ID29 and that of B-93 in the interval between ID71 and RM7029 were in contradiction to their phenotypes (Figure 1A). By excluding these two intervals, we found that *qBlsr5a* is located within a 78.3-kb region between markers ID29 and ID71 (Figure 1A).

**Table 1:** InDel markers developed in this study.

Marker name	Primer sequence (5′→3′)	
	Forward	Reverse
ID11	TTCCCCTTCCTTCGATCTTT	GGCAGACAGTGGCACAAGTA
ID8	ACGATTGACGACGAGCTTCT	ACTCCCTCCGTTTCACAATG
ID29	AATTCGCAAGCTACCGTCAT	CGGGGTCTCGATTGATCTC
ID17	GCATCGCAATGATTGTGTCT	GTGACCCAAAGGACCCCTAC
ID68	TGTACATGCTAGCTGTGCGTA	GAACCCACTCTCCCTCATCA
ID71	GTCGGGAGACGTGAGGAG	GATCTGGTCGGGGAAGTTG
ID48	ATGCCGCTATTATCGATTGG	GTGCCTACCCATATCCGTGT
ID54	CGGACAAGGGAAGAGGAAA	CATTGCCAAAATTTAGTAAGGTTG
ID73	ATCAATAGGTCTCCGGTTCG	CAAATTTTGGGTGTTATTTTGGA
ID79	TCAACTCATTCAATGCAGAACC	ACATATATTCGATCCCTCTGTGC
ID 39	CCTCCCTCCCTCATGTGTAA	TCACTCCCATCTCTGCCTCT
ID45	CCTTATCCGGAACTCCTCCT	GCATCATGTACCCTGGGAAT

To further narrow down the interval of *qBlsr5a*, we developed a new F_2_ population consisting of 4587 seedlings to screen for recombinants between markers ID29 and ID71. Six F_2_ recombinants needed were obtained and corresponding sub-CSSLs were developed in the next (F_3_) generation. Among the 6 sub-CSSLs, two exhibited the H359-BLSR5A genotype at ID29 but the H359 genotype at ID71, while the other four showed the opposite genotypes at the two markers (Figure 1B). Inoculation experiment was performed using a new BLS pathogen strain isolated from the lesions on rice leaves inoculated with the old strain used previously (see above). The new strain showed much stronger pathogenicity, resulting in much longer lesions and therefore enlarging the difference between the two parents (Figure 1B). According to their lesion lengths, the 6 sub-CSSLs could be clearly classified into two groups with 4 sub-CSSLs being susceptible and 2 sub-CSSLs being resistant to BLS, respectively (Figure 1B).

Four new InDel markers (ID73, ID79, ID39 and ID45; Table 1) located between markers ID29 and ID71 were developed and were used, together with the two markers (RM17750 and ID17) already known within this interval, to genotype the 6 sub-CSSLs. The results indicated that the genotype of line A-124 in the interval between ID79 and ID71 and those of B-102 and Hh6 in the interval between ID29 and ID73 were in contradiction to their phenotypes (Figure 1B). By excluding these two intervals, we thus delimited *qBlsr5a* within a 30.0-kb interval between markers ID73 and ID79 (Figure 1B).

### Coding Sequences and Gene Expression at *qBlsr5a*

**Figure 2:**
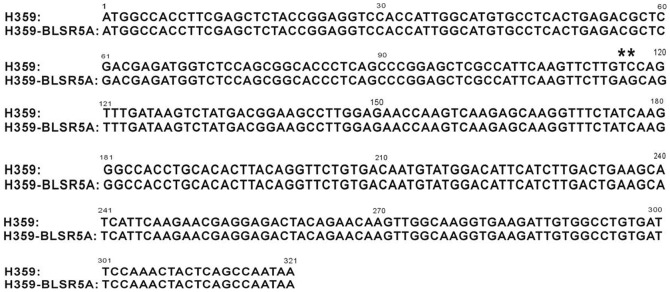
Comparison of the coding sequences of gene LOC_Os05g01710 from the two parents. Asterisks indicate the substituted nucleotides.

According to the Rice Genome Annotation Project (http://rice.plantbiology.msu.edu/), there are three genes predicted in the 30.0-kb interval of *qBlsr5a*, including LOC_Os05g01700 (encoding ABC transporter), LOC_Os05g01710 (also named *TFIIAγ*, encoding transcription initiation factor IIA gamma chain) and LOC_Os05g01730 (also named *Di19*, encoding drought induced 19 protein). Using specific primers (Table 2), the coding sequences of the three putative genes in the two parents were isolated and sequenced. The coding sequences of LOC_Os05g01700 and LOC_Os05g01730 from the two parents were exactly the same and also identical to those from Nipponbare. However, a variation was observed in the coding sequence of LOC_Os05g01710 between the two parents. While the allele from the susceptible parent H359 is identical to that from Nipponbare, which was also found to be susceptible to BLS according to our experiment, there are two successive nucleotides altered in the allele from the resistant parent H359-BLSR5A (Figure 2). The nucleotide alteration results in a change of the 39^th^ amino acid of the TFIIAγ protein from valine (GTC) in H359 to glutamic acid (GAG) in H359-BLSR5A.

**Table 2:** Primers used for coding sequence amplification and qRT-PCR analysis.

TIGR gene ID	Primer sequence (5′→3′)	
	Forward	Reverse
LOC_Os05g01700	TGAGTCTGCGCTTCAAGAAA	TATGCTCATCCCTGCAACTC
	TAGCTGCAGTTGCAGAAGGA	TTCGAGTTGCTGTTCTGTGG
LOC_Os05g01710	TCTGGAATTTGCTCGCGTTC	AAACCCTGACCTCGCAGTTA
	CTATGACGGAAGCCTTGGAG	CAGGCCACAATCTTCACCTT
LOC_Os05g01730	TAGTGAGTGGTGAGAGGGGA	AAAAGCCTACCCAAACTGCC
	GCCCCTTCTGCTACATTGAG	TGTGAGTGCTGAACCCTGAA
Actin	GATTGCCAAGGCTGAGTACGA	AAAAGAGAGAAACAAGCAGGAGGA

Note: For each putative gene, primers for coding sequence amplification are shown in the upper row; primers for qRT-PCR are shown in the lower row. The primers for Actin gene were used for qRT-PCR.

**Figure 3:**
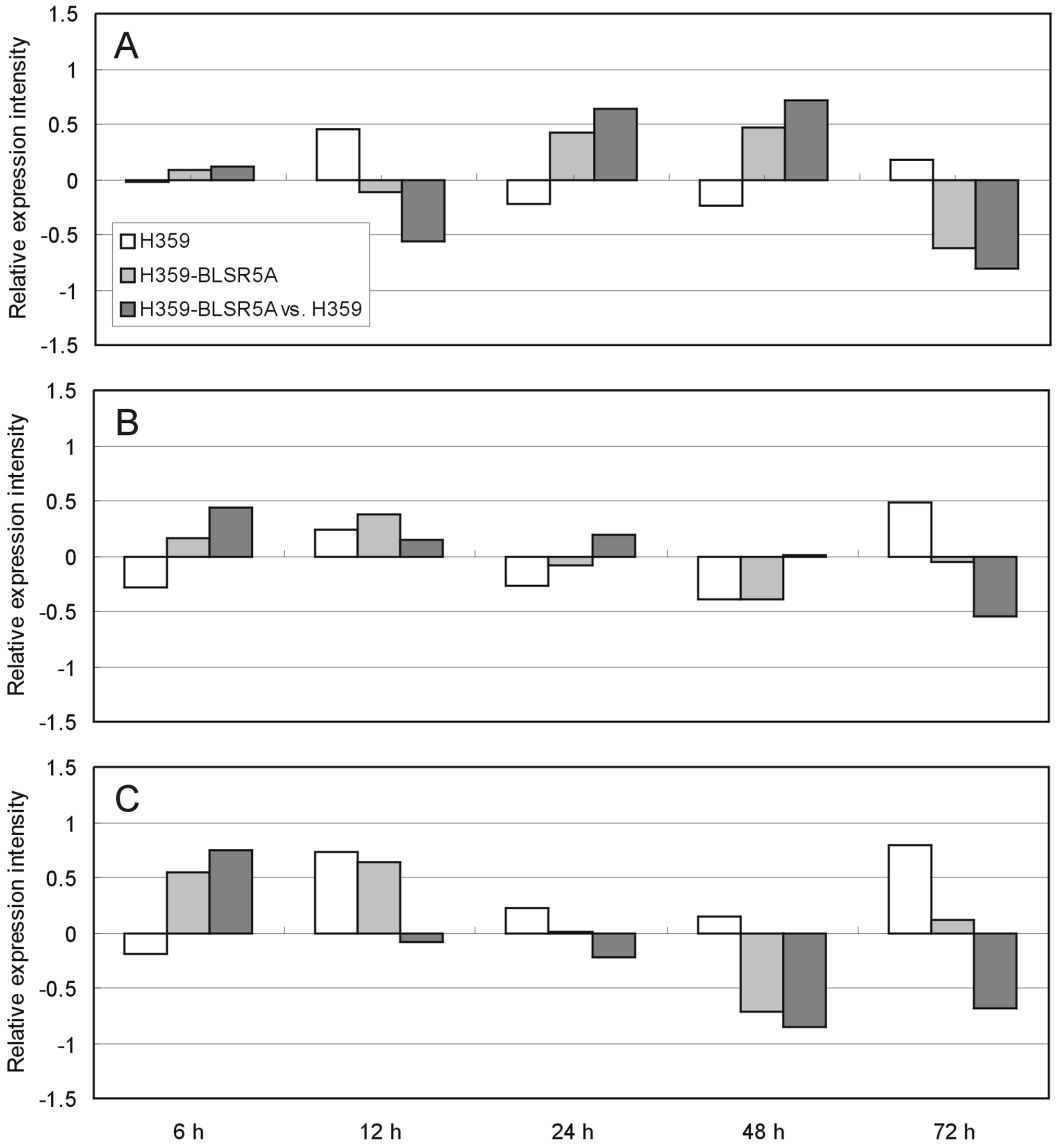
Expression response of LOC_Os05g01700 (A), LOC_Os05g01710 (B) and LOC_Os05g01730 (C) to BLS pathogen in the two parents. The expression is thought to be significantly up−/down-regulated when the relative expression intensity, defined as log_2_[(expression intensity in treatment)/(expression intensity in control)], is greater/smaller than 1/−1.

qRT-PCR analysis using gene-specific primers (Table 2) showed that none of the three predicted genes within the interval of *qBlsr5a* displayed significant response to the inoculation of BLS pathogen in the two parents and significantly different expression patterns between the two parents (Figure 3). These results suggested that gene expression might not be the cause of the genetic effect of *qBlsr5a* on BLS resistance.

## Discussion

### Strategies for Fine Mapping of QTLs

Fine mapping of QTLs is the prerequisite for positional cloning of QTLs and precise marker-assisted selection of QTLs in crop breeding. There are two general principles for fine mapping QTLs. One is to reduce the multigenic inheritance of a quantitative trait into monogenic inheritance by constructing a series of secondary mapping populations, in each of which only one QTL related to the target trait is segregated, so as to farthest reduce the genetic background variation for each target QTL. The other is to measure quantitative traits based on lines instead of individuals so as to reduce the effects of environmental variation, unless the target QTL has very large effects. Following these principles, many QTLs have been fine mapped or even cloned in plants using various experimental strategies [27]−[33]. However, the number of fine mapped QTLs conferring disease resistance in plants, especially those with small effects, is still very limited.

In this study, we fine mapped the QTL *qBlsr5a* conferring BLS resistance in rice by developing a set of overlapping sub-CSSLs across the target QTL region, narrowing down the QTL interval from ∼380 kb to 30.0-kb. The strategy used in this study is similar to that used by Brouwer and St. Clair [34] in principle, with which they fine mapped three quantitative trait loci for late blight resistance in tomato (note: in their paper, CSSL is termed as near isogenic line or NIL and sub-CSSL as sub-NIL). Phenotyping is a main limiting factor in QTL fine mapping, especially for quantitative disease resistance [11]. Our study demonstrated that the strategy we used provides an effective solution for the phenotyping of quantitative disease resistance on both accuracy and efficiency, and is therefore quite suitable for the fine mapping of disease resistance QTLs. First, most disease resistance QTLs have small effects. Using sub-CSSLs can effectively reduce the environmental variation and therefore obtain more accurate phenotypic observations. Second, measuring plant resistance to disease is generally very laborious. Using sub-CSSLs can dramatically reduce the workload of phenotyping, since they are developed from recombinant plants, which only account for a very small proportion in a secondary population. In addition, disease resistance QTLs often have no or only partial dominance effects. For example, the QTL analyzed in this study showed almost no dominance effect (the mean length of BLS lesions in the F_1_ of H359×H359-BLSR5A was 1.08±0.21, very close the mid-parent value; Figure 1A). Hence, QTL detection had better be performed based on additive instead of dominance effects so as to achieve higher statistical power. Using sub-CSSLs can meet this requirement because they are homozygous lines.

Apart from being generated from a cross between a CSSL and the recurrent parent as in this study, a secondary population can also be developed from advanced backcross generations (e.g. BC_3_F_1_ or BC_4_F_1_) by selfing selected plants that have got the recurrent parent genetic background but carry an introgressed segment harboring the target QTL from the donor parent, just equivalent to the F_1_ between a CSSL and the recurrent parent. By selecting multiple desirable plants and asexually reproducing the selected plants, it is possible to produce a sufficiently large secondary population for fine mapping the target QTL. Thus, it is possible to complete the fine mapping of a QTL within 5 or 6 generations (from F_1_ to BC_3_F_3_ or BC_4_F_3_), significantly speeding up the process of QTL fine mapping.

Recently, Yang et al. [35] and Zhang et al. [36] reported their work of fine mapping of a major QTL *qRfg1* and a minor QTL *qRfg2* conferring resistance to *Gibberella* stalk rot in maize, respectively. They adopted a so-called step-by-step (or more legibly, generation-by-generation) narrowing-down strategy, in which the fine mapping process was performed based on successive backcross generations, beginning from BC_4_F_1_ till BC_6_F_1_ (for *qRfg1*) or BC_8_F_1_ (for *qRfg2*). In each generation, recombinants were identified and further backcrossed, and their resistance phenotypes were measured based on their backcross progeny lines. Their results indicated that this strategy is applicable to both major and minor QTLs for disease resistance. However, this strategy appears not quite suitable for self-pollinated plants (e.g. rice) because producing a large backcross population in self-pollinated plants is often very laborious. In addition, unlike sub-CSSLs, half of the backcross progeny lines are segregated within the family. Therefore, progeny lines with larger size are required to detect the target QTL effect.

### The Candidate Gene of *qBlsr5a*

We have seen that there are three putative genes predicted in the interval of *qBlsr5a* according to the Rice Genome Annotation Project (http://rice.plantbiology.msu.edu/). LOC_Os05g01700 is predicted to be involved in the biological process of fatty acid beta-oxidation and response to stress. In addition, it possesses the same domain as that in wheat gene *Lr34*, which confers broad-spectrum and durable resistance in wheat [14]. LOC_Os05g01710 is predicted to function in the transcription initiation from RNA polymerase II promoter and be involved in the defense response to bacteria. LOC_Os05g01730 is predicted to respond to water deprivation and be involved in blue and red light signaling pathway. Based on these annotations, it appears that LOC_Os05g01700 and LOC_Os05g01710 are more possibly related to BLS resistance. Since all of the three genes did not exhibit obvious response to BLS pathogen infection (Figure 3) and allelic difference between the two parents was found only in the coding sequence of LOC_Os05g01710 (Figure 2), it can be further inferred that LOC_Os05g01710 is more likely to be the candidate gene of *qBlsr5a*.

Interestingly, sequence comparison indicated that the allele of LOC_Os05g01710 from the resistant parent H359-BLSR5A is exactly the same as *xa5*, which is a major gene resistant to bacterial leaf blight (BLB), another important bacterial disease of rice [8], [37]. In fact, a major gene for BLB resistance was once mapped in this region based on a recombinant inbred line population derived from a cross between H359 and Acc8558 [38]. The result of this study clarified that the resistance gene was actually *xa5*. Meanwhile, the result of this study also suggested that *xa5* might also contribute to the BLS resistance. Considering that BLS and BLB are both bacterial diseases, the putative pleiotropy of *xa5* should not be very surprising. The molecular function of TFIIAγ, which is a component of a general transcription factor, may provide a reasonable explanation for the pleiotropy of *xa5*. In addition, it may also explain why LOC_Os05g01710 did not show apparent response to the infection of BLS pathogen and has the similar expression pattern in the two parents. Actually, *xa5* was also found not responsive to the infection of BLB pathogen [8].

Since *qBlsr5a* only exhibits additive effect but no dominance effect and the effect is small, it is not feasible to confirm the candidate gene of *qBlsr5a* by means of genetic complementation test. A possible way to validate the candidate gene of *qBlsr5a* is to examine whether the BLS resistance level is increased in the transgenic plants of the susceptible parent H359 in which the resistant allele of the candidate is overexpressed, or decreased in the transgenic plants of the resistant parent H359-BLSR5A in which the susceptible allele of the candidate is overexpressed. This work is being on the way.
